# Evolution of Influenza A Virus by Mutation and Re-Assortment

**DOI:** 10.3390/ijms18081650

**Published:** 2017-08-07

**Authors:** Wenhan Shao, Xinxin Li, Mohsan Ullah Goraya, Song Wang, Ji-Long Chen

**Affiliations:** 1 Key Laboratory of Fujian-Taiwan Animal Pathogen Biology, College of Animal Sciences, Fujian Agriculture and Forestry University, Fuzhou 350002, China; ssjwen@outlook.com (W.S.); xinxinlfjfz@163.com (X.L.); goraya_uaf@yahoo.com (M.U.G.); 2 CAS Key Laboratory of Pathogenic Microbiology and Immunology, Institute of Microbiology, Chinese Academy of Sciences, Beijing 100101, China

**Keywords:** influenza A virus, mutation, re-assortment, virus-host interaction

## Abstract

Influenza A virus (IAV), a highly infectious respiratory pathogen, has continued to be a significant threat to global public health. To complete their life cycle, influenza viruses have evolved multiple strategies to interact with a host. A large number of studies have revealed that the evolution of influenza A virus is mainly mediated through the mutation of the virus itself and the re-assortment of viral genomes derived from various strains. The evolution of influenza A virus through these mechanisms causes worldwide annual epidemics and occasional pandemics. Importantly, influenza A virus can evolve from an animal infected pathogen to a human infected pathogen. The highly pathogenic influenza virus has resulted in stupendous economic losses due to its morbidity and mortality both in human and animals. Influenza viruses fall into a category of viruses that can cause zoonotic infection with stable adaptation to human, leading to sustained horizontal transmission. The rapid mutations of influenza A virus result in the loss of vaccine optimal efficacy, and challenge the complete eradication of the virus. In this review, we highlight the current understanding of influenza A virus evolution caused by the mutation and re-assortment of viral genomes. In addition, we discuss the specific mechanisms by which the virus evolves.

## 1. Introduction

In both the northern and southern parts of the world, the outbreaks of influenza occur mainly in winter, while in areas around the equator outbreaks may occur at any time of the year. Typically, influenza is transmitted from infected mammals through the air by cough or sneezing, and also from infected birds through their droppings. Influenza virus can also be transmitted through saliva, nasal secretions, feces, and blood of the infected animals. In humans, the common symptoms of influenza virus infection are fever, muscle pain, severe headache, coughing, sore throat, weakness, and fatigue.

Influenza virus, which belongs to the family of Orthomyxoviridae, consists of eight segments of negative-sense single-stranded RNA. The orthomyxoviridae is further divided into five genera: influenza A virus (IAV), influenza B virus, influenza C virus, isavirus, and thogotovirus [1]. Influenza B and C viruses only infect humans, causing the respiratory disease [2,3]. IAV poses a significant risk of zoonotic infection, host switch, and the generation of pandemic viruses. IAVs can infect humans and a variety of animals, such as pigs, horses, marine mammals, cats, dogs, and birds. Although the history of IAV seems to originate from ancient Greek in about 413 BC [4], the first confirmed pandemic was the Russian flu that occurred in 1889–1892 [5].

There are three main characteristics which contribute to the rapid evolution of these viruses: large populations, short generation times, and high mutation rates. Every mutation, which helps the virus to evade the host immune system, may be positively selected, passed on to the next generation, and distributed more widely. Over the past 100 years, there have been four flu pandemics: 1918 H1N1 Spanish flu [6], which has been described as "the greatest medical holocaust in history" [7], 1957 H2N2 Asian flu, 1968 H3N2 Hong Kong flu, and 2009 H1N1 swine flu. The most serious pandemic occurred in 1918, killing more than 50 million people worldwide [8]. Since 1996, the highly pathogenic avian influenza (HPAI) virus (H5N1) has caused more than 1000 deaths, resulting in a mortality rate of about 55% [9]. Recently, a low-pathogenic avian influenza (LPAI) virus (H7N9) identified first in the East China region caused an outbreak in humans, with a mortality rate as high as 40% [10].

## 2. Classification of the Virus

The influenza virus genome is about 13kb and encodes 13 proteins [11]: Hemagglutinin (HA), neuraminidase (NA), M1 matrix protein (M1), M2 ion channel protein (M2), nuclear protein (NP), nonstructural protein (NS1, NS2), and RNA polymerase complex (PB1, PB2, PA) [12]. On the basis of their surface glycoproteins, HA and NA, influenza A viruses can be divided into 18 HA subtypes and 11 NA subtypes, and further classified by strains, while influenza B and C viruses are classified only by strains [3,13].

The IAV genes encoding the viral surface proteins, HA and NA, that form the main targets of neutralizing antibodies, are critical for the evolution of the virus. HA is a trimeric glycoprotein, and each monomer has two polypeptide chains: HA1 and HA2. There are 16 classical subtypes of HA which are classified in two groups and four clades: group 1 contains the H1 clade (H1, H2, H5, H6, H11, H13, H16) and the H9 clade (H8, H9, H12), while group 2 includes the H3 clade (H3, H4, H14) and the H7 clade (H7, H10, H15) [14,15]. Recently, two more HA subtypes have been discovered exclusively in bats (H17 and H18) [16]. NA is a tetramer of four identical polypeptide chains. The nine classical subtypes of NA are divided into three groups: Group 1 comprises N1, N4, N5, N8, group 2 comprises N2, N3, N6, N7, N9, and group 3 contains NA of influenza B viruses. Recently, two new NA subtypes have been found in bats (N10 and N11) [16]. Human influenza viruses have only limited subtypes of HA and NA, including H1, H2, H3 and N1, N2, whereas influenza viruses that infect poultry may include almost all subtypes of HA and NA [17].

## 3. IAV Life Cycle and Host Antiviral Response

IAV membrane protein HA plays an important role in the establishment of viral infection. The influenza virus binds to the sialic acid on the cellular glycoprotein or glycolipid through the outer side of the head of the HA molecule. Specific sialic acid linkages and penultimate galactose determine the host specificity of the virus. For example, the HA of human adapted influenza virus preferentially recognizes receptors with a terminal α-2,6 sialic acid (SA), whereas the avian IAV preferentially recognizes receptors with a terminal α-2,3 SA [11,18]. The α-2,6 SA are found on bronchial epithelial cells of the human upper respiratory tract (URT) and the α-2,3 SA are found on epithelial cells of the birds’ intestine and on the lower respiratory tract (LRT) of humans [15]. Thus, differences of cell surface receptors constitute a major barrier to cross-species transmission of influenza virus. HA is a HA1-HA2 dimer linked by disulfide bonds formed by the hydrolysis of HA0 precursor [19]. HA1 has a functional domain that binds to the host cell receptor, and HA2 is an important subunit that mediates the fusion of the virus with the host cell membrane. Therefore, influenza virus enters the host cell through HA1-mediated binding with the cell receptor, followed by HA2-mediated virus fusion with the cellular membrane and finally the invasion of host cells [20].

The cleavage of the IAV particles from the binding site of the host cell surface is mediated by the action of the viral NA [19]. NA has an enzymatic activity to promote the dispersion of influenza virus in respiratory mucosal secretions [21]. It is important that NA enzymatic activity removes sialic acid from viruses and cellular glycoproteins to facilitate the release of progeny virus particles from cells and then infect the other cells [22,23,24]. Because HA and NA are responsible for the binding to and the release from host cell receptors, they are crucial in determining host specificity [25]. The virulence of IAVs is largely dependent on the coordination of HA and NA. It has been observed that the HA/NA balance is one of the key factors in the transmission of NA inhibitor-resistant strains in the human population [26].

Unlike many other RNA viruses, the replication and transcription of influenza viruses are carried out in the nucleus of the host cell. Within the influenza virus particles, the NP binds to the viral RNA gene segment and the viral RNA polymerase complex binds to the end of the viral RNA, and they together form the vRNP containing the RNA-protein complex [27]. Influenza virus vRNP is the basic replication unit of influenza virus, composed by the virus RNA and four kinds of viral proteins including NP, PB1, PB2, and PA [28,29]. PB1, PB2, and PA together constitute an RNA polymerase complex that is critically involved in the genomic replication and expression of influenza virus [29].

In addition, the pathogenicity of IAV is associated with the host immunity [30,31]. IAV infection induces host innate immune responses, which can result in the termination of the virus life cycle. On the other hand, influenza virus has evolved multiple strategies to circumvent the host’s innate immunity to establish a successful infection and replication [32,33]. The initial innate immune responses to viral infection are the release of various cytokines induced by immune signaling pathways. Influenza virus RNA is detected by host pattern recognition receptors (PRRs) and is very effective in initiating the activation of innate signaling pathways. The viral pathogen-associated molecular patterns (PAMPs) such as IAV RNA are recognized as foreign by PRRs including toll-like receptors (TLRs), retinoic acid-inducible gene I (RIG-I)-like receptors (RLRs), and nucleotide oligomerization domain (NOD)-like receptor family pyrin domain containing 3 (NLRP3), which triggers the expression of type I and type III interferons (IFNs) [34,35], pro-inflammatory cytokines, and chemokines [32,36]. Moreover, IAV infection can attract alveolar macrophages, monocytes, and natural killer (NK) cells. However, an overly aggressive innate response, such as the excessive production of cytokines with early recruitment of inflammatory leukocytes to the lung, is believed to contribute to the pathogenesis of influenza virus. For example, it has established that the dramatic fluctuations in the release of cytokines lead to inflammation and organ damage, which are the cause of acute clinical symptoms [34,37,38].

## 4. IAV Evolution and Underlying Mechanism

It is well known that the influenza viral RNA-polymerase represents the lack of proofreading function. Thus, the integration of faulty nucleotides often occurs during the viral replication process with a rate of 10^−3^ to 10^−4^, which results in high mutation rates [39,40].

Due to its crucial role in receptor recognition and attachment, IAV HA is considered to be a principal determinant of the host-range. The specificity of the HA of avian influenza viruses is for α-2,3 SA receptors found in the intestinal tract of the bird, whereas α-2,6 SA receptors are predominantly found in the upper respiratory tract of humans. Recently, it has been shown that mutations in the HA protein alter its receptor-binding preference that allows the highly pathogenic avian H5N1 IAV to transmit between mammals [41]. Therefore, it is not surprising that multiple changes in gene segments of the avian influenza virus could result in its adaptation to humans [1]. On the other hand, owing to having both α-2,3 and α-2,6 linkages, pigs and several avian species (pheasants, turkeys, quails) may act as mixing vessels and can generate re-assortment viruses [42,43].

Influenza proteins must evade immune recognition while maintaining their ability to function and interact with host cellular factors [44]. The three mechanisms by which influenza viruses undergo evolutionary change include mutation (antigenic drift), re-assortment (antigenic shift), and, in rare instances, recombination. The different virus lineages are predominantly host specific, but there are periodic exchanges of influenza virus gene segments between species, giving rise to pandemics of disease in humans, lower animals, and birds [45]. Influenza virus evolution proceeds via re-assortment and mutation, and such evolution can influence the host specificity and pathogenicity of these viruses [46]. Genetic variations of influenza A virus lead to possible changes in upcoming epidemiological behavior and may result in human pandemics.

### 4.1. Mutations

Significant mutations in antigenic sites resulting from constant point mutations in the influenza virus contribute to the gradual evolution of the virus, leading to antigen migration to produce new influenza virus subtypes to escape the immune pressure of the population [47]. All subtypes of influenza A virus antigenic drift can occur, but such antigenic drift often occurs in the general human influenza. Immune escape can be achieved by mutation in IAV proteins such as HA and/or NA. The minimal structural changes can occur in these surface proteins and so the immune protection of the host (acquired through previous infections or immunization) will no longer be effective against the invading virus. As a consequence, the immune system is unable to identify the newly changed virus variants and the recognition pattern of the antigen-antibody-interaction is not fully functional anymore. In addition, amino acid substitutions in HA protein can change the receptor preference of influenza virus. Some studies have shown that the G186V mutation in HA protein was noted as a potential adaptation of avian H7 to human-type receptors [48,49]. In A/Vietnam/1203/2004 (H5N1) virus, K58I substitution in HA protein is associated with increased viral replication of upper respiratory tracts in mice and ferrets [50]. Remarkably, the K58I substitution combined with a G219S mutation in HA protein increased the overall affinities of binding to α-2,3 and α-2,6 SA of the A/Anhui/1/13 (H7N9) virus [51]. Furthermore, there is a R292K mutation in NA protein in H7N9 virus strains which had been isolated from a patient after drug treatment. This substitution was found to promote drug resistance; in particular, it gave a high resistance to oseltamivir which is the most commonly used anti-influenza drug [52]. Antigenic drifts are the main reason for new variants and cause annual influenza outbreaks. Although these changes may not lead to pandemics, antigenic drift over a period of time can make a strain considerably different from the original pandemic virus.

It has been confirmed that the long-term evolution of cytotoxic T lymphocyte (CTL) epitopes is associated with CTL-mediated clearance of infection and it is thought that the selection pressures imposed by CTL immunity shape the long-term evolution of IAV [53,54]. Viruses mutate amino acid residues within CTL epitopes to evade CTL recognition [55]. Under certain circumstances, amino acid substitutions occur at the anchoring residues, while in other cases they occur at the T cell receptor contact residues [56]. For instance, mutations at the anchored residues of the CTL epitope have been described in the human leukocyte antigen (HLA)-B* 2705 restricted NP_383–391_ epitope, which has the R-to-G substitution at position 384 (R384G) [57,58]. This replacement significantly reduced the in vitro virus-specific CTL response in HLA-B* 2705-positive individuals.

Moreover, it has been found that the E627K mutation of PB2 enables the replication of influenza virus carrying PB2 from a poultry source to human respiratory epithelial cells [59]. Interestingly, it has been suggested that the PB2 gene of the 2009 influenza A virus (H1N1) may mutate at another amino acid site under the immune pressure, and replace the effect of the E627K mutation, which significantly enhances the adaptability of the 2009 H1N1 influenza virus in the human population [60]. Human seasonal influenza virus strains and human pathogenic H5N1, H7N9, and H10N8 viruses are common in the 627-K PB2 mutation [61,62]. Studies have shown that duck 3286/H7N9 virus PB2 gene mutation enhances polymerase activity and viral replication in human cells [63]. The data reveal that the E627K substitution in the PB2 polymerase protein appears to be one of main determinants of the avian influenza virus host range [64].

K526R mutation in PB2 is a new adaptive marker for the human H7N9 and H5N1 (Indonesia) avian influenza A viruses, and provides additional benefits for H3N2 virus replication. Previous studies observed that the substitution of 526R in PB2 enhanced RNP polymerase activity by coordination with NEP expression, and thereby promoted the viral transcription and genomic replication during the IAV life cycle [65]. In addition, the H1N1 subtype virus that caused the 1918 influenza pandemic as well as the H5N1 avian influenza virus identified in Hong Kong in 1997 are both highly pathogenic influenza viruses and contain N66S mutation in PB1-F2. It was suggested that this mutation significantly enhanced the viral pathogenicity of these viruses [66].

### 4.2. Re-Assortment

It has been well recognized that the segmented genome of the influenza virus allows the exchange of RNA segments between genotypically different influenza viruses, resulting in the production of new strains and/or subtypes [67], which is referred to as re-assortment. A pandemic IAV can be produced by transmission from animals to humans or by reconfiguration between avian influenza viruses and human influenza viruses [68]. As the influenza virus has a segmented genome, re-assortment is an important mechanism for generation of the "novel" virus [69]. Thus, re-assortment of the virus achieves a new antigenic pattern known as "antigenic shift". Pandemic influenza emerges as a result of such major genetic changes of IAV. These modifications occur due to mechanistic errors during the replication of viral RNA polymerase, evolutionary pressure, the novel environment of the host, immune pressure, or antiviral drug pressure [70]. Two of the three major human influenza pandemics in the twentieth century (1957 and 1968) and this century (2009) were due to the re-assortment between the human IAV and other host species.

There is evidence indicating that the HA, NA, and PB1 genes of the H2N2 1957 pandemic strain in addition to the HA and PB1 fragments of the H3N2 1968 pandemic strain are both avian, and the remaining fragments may come directly from 1918 [67]. The first influenza pandemic in this century, the influenza A H1N1 virus, is a re-assortant caused by a multiple mixed recombination between the European H1N1 swine influenza virus, North American H1N2 swine influenza virus, North American avian influenza virus, and H3N2 influenza virus [71]. This re-assortment includes PB2, PA from the North American swine influenza virus, PB1 from human H3N2 influenza virus, HA, NP, NS from a classic swine influenza virus, and NA, M from the European H1N1 swine influenza virus [72]. In 1996, the HPAI virus subtype H5N1 was isolated from geese in Guangdong province (Gs/GD) [73]. This virus evolved into a variety of HA genetic progeny and was reclassified with different NA and internal genes to produce subtype H5N8 clades 2.3.4.4 Gs/GD HPAIV. It first appeared in poultry outbreaks in 2013 [74], followed by the outbreak of South Korea in January 2014 [75]. At the end of 2014, the HPAI H5N8 virus also invaded several countries in Europe [76], and in the summer of 2016, novel HPAIV (H5N8) was detected in wild aquatic birds sampled in western Siberia [73]. In May 2016, the H5N8 virus strain caused deaths among three species of wild migratory birds in Qinghai Lake, China. The study showed that the novel re-assortant virus belongs to group B H5N8 viruses and the re-assortment events likely occurred in early 2016 by genetic analysis. Thus, with wild birds migrating, the H5N8 virus may have spread to other areas along the flight [77]. In addition, the re-assortment between the Gs/GD lineage H5N8 virus and North American origin viruses resulted in the emergence of H5N1 and H5N2 viruses in the US [78]. The novel re-assortant H5 HPAI clade 2.3.4.4 virus (H5N2) caused an outbreak in poultry farms in British Columbia, Canada, during November 2014. It was subsequently discovered in the US in wild waterfowl, raptors, and backyard poultry, including domestic ducks and geese [79,80].

### 4.3. Recombination

In addition to mutation and re-assortment, IAVs still have another relatively rare means of evolution called recombination. Genetic recombination is one of the primary processes that produce the genetic diversity upon which natural selection acts. Recombination in IAVs can occur through two main mechanisms: one is the non-homologous recombination that occurs between two different RNA fragments [81,82]; the other is the controversial homologous recombination, often considered to be absent or very rare, which is thought to participate in template switching while the polymerase is copying the RNA. During IAV replication, the generated genomic RNA is rapidly packaged with ribonucleoprotein that is involved in preventing the occurrence of template-switching. Therefore, homologous recombination is thought to be rare in influenza viruses [83]. However, there are studies claiming that controversial homologous recombination may exist during IAV replication. For example, a previous study indicated that homologous recombination can occur in PB2 and PA, HA (between human H1N1 subtype strains) and NP (between human H1N1 subtype strain and H3N2 subtype strain) [84]. In addition, recombination between divergent variants has generated new genotypes and may play a crucial role in the evolution of H5N1 viruses [85]. The current study confirmed that the PB1 segment of the novel H7N9 might have been characterized by recombination. At the site of 490–780nt, a fragment derived from an HPAIV strain, A/tree sparrow/Thailand/VSMU-16-RBR/2005 (H5N1), has been recombined into its PB1 segment. It is likely that the recombinant PB1 segment appeared in the form of H9N2 or H5N1 in about 2007, before being re-assorted into the novel H7N9 which caused an epidemic in 2013 [86]. Other studies have also provided homologous recombination evidence in human and swine IAVs [84,87]. It is worth noting that recombination contributing to the generation of genetic diversity can only occur among viruses that replicate within the same cells.

## 5. The Risk of IAV Evolution

Wild waterfowl and shorebirds belong to the main natural host species of IAV [88]. IAV has been able to establish the successful infection of a variety of animals, including avian and mammalian species, and its evolution has led to the emergence of IAV in human beings for a long time [89]. Since the pandemic outbreak of influenza virus in 1918, the re-assortment of influenza virus has occurred among bird and human viruses. As described above, the re-assortment of influenza viruses has resulted in the pandemic of H2N2 in 1957 and of H3N2 in 1968 [90]. During the year 2009, there was an outbreak of H1N1 in humans that caused the first pandemic of influenza through human transmission in the 21st century [91].

Usually, an avian influenza subtype does not infect humans and a human influenza subtype is unable to infect the birds. However, swine acts as a virus mixer vessel, leading to the generation of new influenza viruses, which can infect both humans and poultry. The mutation and re-assortment of the IAV genome are susceptible to forming new subtypes of influenza virus that may result in widely propagated and destructive pandemics due to the lack of immunity to the emerging pathogen [67]. For example, the outbreak of H5N1 avian influenza in 1997 and the outbreak of H1N1 swine influenza in 2009 caused great panic and brought serious economic losses to the breeding industry.

The influenza virus can be propagated by the direct carrying of the virus, through animal feces, or via the pollution of water. Such propagation can affect a wide range of hosts, including human, livestock, and birds. Importantly, infected wild birds and poultry can be the major source of virus dissemination. Notably, the wide communication of influenza virus significantly increases the odds of genetic mutation and re-assortment [62]. Simultaneously, the variation of the virus caused by genetic mutation and re-assortment also has a serious impact on human and animals.

Since the avian flu pandemic in 2013, H7N9 has achieved remarkable results in effectively overcoming species barriers as it spread between poultry and humans, and it is estimated to be unpredictably communicated between poultry and humans. According to the World Health Organization (WHO) statistics, there are at least 779 human deaths, and the death rate is gradually rising every year. Chen et al. found that the initial outbreak of H7N9 in the live poultry market was affected by infected poultry [92].

However, to date, the related data show that all strains causing a human pandemic have illustrated the specificity in human receptors, which includes the pandemic H3N2 strain in 1968, the pandemic H1N1 strain in 1977 and 2001, and the pandemic H1N1 strain in 2009 [93]. Similarly, humans can be infected with an avian influenza virus in the absence of a receptor-specific influenza virus. To a certain extent, H7N9, with genetic mutation, is related to the Q226 receptor mutation in the HA protein which could maintain the receptor specificity in avian receptor [94]. Simultaneously, combined with other receptor mutations, it will stimulate the specificity of human receptors and increase the binding rate of human receptors, thus upgrading the prevalence of human disease. In addition, Schrauwen et al. inferred that there are more mutations that may be completely transformed into human-specific receptors [51]. These studies have achieved a breakthrough in the spread of avian influenza in human beings. The link between the number of H7N9 HA mutations and their pathogenicity has also been studied by de Vries et.al, who found that recombinant H7 proteins need three amino acid mutations to change specificity to human-type receptors [93]. In 2015, a case of severe pneumonia infected by H7N9 occurred. Studies found that the HA gene and NA gene had a higher homology compared with the H5N6 subtype AIV, while the other six genes were not as highly similar as the HA gene and NA gene.

The continuous infection and propagation of the virus could bring out genetic mutation or genetic re-assortment and also can affect pathogenicity and virulence. Studies found that the low pathogenic avian influenza virus strains (H9N2), which produce the rapid spread of the virus among poultry for 6 to 9 months, could become HPAI virus strains, leading to great economic losses to the poultry industry. It was explained that H9N2 could infect humans by its internal gene generating re-assortment with other subtypes of influenza, such as the subtypes of H5N6, H7N9, and H10N8 [95], which all infected humans in combination with H9N2. In another study, it was proven by genetic analysis that the H5N6 subtype isolated in Yunnan was a re-assortment of the H9N2 virus and H5N6 virus. Notably, H9N2 has been transmitted to Egypt recently and, since its occurrence in Egypt, the number of people infected with H5N6 influenza and the number of H5N6 avian influenza cases in Egypt have been increasing regularly [96].

## 6. Conclusions

Influenza is still a top threat to global public health; 250,000~500,000 people die from the influenza every year throughout the world [97]. WHO annually conducts global surveillance of influenza, and predicts several types of representative viruses that may be popular. According to the surveillance results, seasonal IAV vaccines are annually reformulated, manufactured, and delivered for administration to global human population just before the start of flu season [98]. To date, vaccination is the staple method to decrease epidemical influenza. For example, attenuated vaccines and trivalent inactivated vaccines that may include three inactivated or weakened influenza viruses of selected H1N1 and B-type viruses can effectively reduce and prevent infection by IAV and alleviate flu symptoms [99,100]. However, the lag time between virus identification and vaccine distribution exceeds 6 months. Thus, the vaccine is unable to provide immediate protection against sudden influenza outbreaks.

Now, in addition to vaccination, the development of antiviral agents with immunomodulatory characteristics is very important to combat pandemic influenza virus strains. Both conserved viral proteins and host proteins may be applied as targets in order to achieve broad antiviral activity. Various novel antiviral agents have been developed for existing targets M2 and NA or other IAV components, and several of them have shown promising results in clinical trials [101]. Furthermore, viral RNA is also being targeted by antisense and short-interfering RNAs to develop alternative anti-IAV therapeutics [102]. Therefore, novel approaches that have both immunomodulatory and antiviral effects deserve special attention [97]. Finally, host factors involved in IAV replication and pathogenesis are also explored as targets for the development of anti-IAV strategies [103].

Apart from continuous surveillance, the development of universal vaccines and effective antivirals as well as the effective management of hosts is required to restrict the circulation and generation of new and more virulent IAV variants. It is necessary to study the molecular mechanisms that may explain why some animals are resistant to IAV infection, and then identify genes that contribute to such resistance. Afterward, the intermediate host may potentially be made resistant to IAV infection by inserting those genes or using RNA interference and CRISPR-Cas (Clustered Regularly Interspaced Short Palindromic Repeats/CRISPR-associated genes) methods [104,105]. Although various strategies to prevent influenza have been developed, there is still a long way to go in order to eradicate influenza virus because of its continuous evolution.
